# Stigma, social reciprocity and exclusion of HIV/AIDS patients with illicit drug histories: A study of Thai nurses' attitudes

**DOI:** 10.1186/1477-7517-5-28

**Published:** 2008-08-23

**Authors:** Kit Yee Chan, Mark A Stoové, Daniel D Reidpath

**Affiliations:** 1 Nossal Institute for Global Health, University of Melbourne, Melbourne, Victoria, Australia; 2 Centre for Population Health, Macfarlane Burnet Institute for Medical Research and Public Health, Melbourne, Victoria, Australia; 3 Centre for Public Health Research, Brunel University, London, UK

## Abstract

**Background:**

Stigma is a key barrier for the delivery of care to patients living with HIV/AIDS (PLWHA). In the Asia region, the HIV/AIDS epidemic has disproportionately affected socially marginalised groups, in particular, injecting drug users. The effect of the stigmatising attitudes towards injecting drug users on perceptions of PLWHA within the health care contexts has not been thoroughly explored, and typically neglected in terms of stigma intervention.

**Methods:**

Semi-structured interviews were conducted with a group of twenty Thai trainee and qualified nurses. Drawing upon the idea of 'social reciprocity', this paper examines the constructions of injecting drug users and PLWHA by a group of Thai nurses. Narratives were explored with a focus on how participants' views concerning the high-risk behaviour of injecting drug use might influence their attitudes towards PLWHA.

**Results:**

The analysis shows that active efforts were made by participants to separate their views of patients living with HIV/AIDS from injecting drug users. While the former were depicted as patients worthy of social support and inclusion, the latter were excluded on the basis that they were perceived as irresponsible 'social cheaters' who pose severe social and economic harm to the community. Absent in the narratives were references to wider socio-political and epidemiological factors related to drug use and needle sharing that expose injecting drug users to risk; these behaviours were constructed as individual choices, allowing HIV positive drug users to be blamed for their seropositive status. These attitudes could potentially have indirect negative implications on the nurses' opinions of patients living with HIV/AIDS more generally.

**Conclusion:**

Decreasing the stigma associated with illicit drugs might play crucial role in improving attitudes towards patients living with HIV/AIDS. Providing health workers with a broader understanding of risk behaviours and redirecting government injecting drug policy to harm reduction are discussed as some of the ways for stigma intervention to move forward.

## Background

Recent studies in Thailand have shown that the stigmatisation of people living with HIV/AIDS (PLWHA) in health care settings can pose a significant barrier to the quality of patient care [1,2]. This makes understanding the nature of the stigma an integral part of a comprehensive approach to the delivery of appropriate treatment and care to PLWHA [3,4]. Stigma is closely related to notions of social exclusion; something often characterised in terms of one group ensuring privilege over another through social processes that separate and distinguish between those groups that are *fit *to contribute and share community resources and those that are not [5-7]. Conceived in these terms, stigma is the symbolic tagging of individuals or groups as sufficiently deviant from a social norm so as to legitimise their exclusion from community membership and social investment [5]. The term social exclusion could, thus, be regarded as an alternative way of describing the discriminatory responses arising out of the process of stigmatisation [8].

One possible reason for the social exclusion of PLWHA could be the perceived social cost associated with their inclusion. In an adaptation of an idea of Robert Trivers [9], we recently suggested that the concept of reciprocity is useful for understanding the relationships between stigma and social exclusion as it allows the relationship between them to be understood in terms of the same social processes involved in controlling the distribution of limited resources [5]. In essence, reciprocal exchange occurs both between individuals and at the broader levels of society. The process has been regarded as a critical process that assisted in bringing society and culture into existence [e.g. [10]]. For example, as individuals draw upon societal resources (e.g. healthcare, education), they have a moral obligation to contribute to those resources through taxation and other means. Failure to reciprocate violates that social trust and indicates a high cost to the community of maintaining a person or group's opportunity to draw on shared resources. In the interests of the community, groups or individuals who are marked as poor reciprocators (i.e. stigmatised) are then excluded from routine social processes. A number of conditions could lead groups and individuals to be judged too costly for social inclusion [[5], p.481]:

1. The potential recipient of the resources is regarded as a 'cheater'; i.e. someone who takes from the society but chooses never to repay the social debt;

2. The potential recipient of the resource is regarded as incapable; i.e. no matter how well intentioned, they may lack the wherewithal to repay the social debt; or

3. As a variation of point two, the potential recipient of the resource is regarded as too transient; i.e. they may wish to repay the social debt but they will die or relocate out of the community before they have a chance.

It should be noted that while reciprocity has important social functions, our judgements of who might be worthy of social inclusion are often arbitrary. Since the mere act of being socially excluded could prevent otherwise able and willing individuals and groups from making social contributions; the perception that the excluded are poor reciprocators could be a self-fulfilling hypothesis that contributes to the suffering of the stigmatised.

The last two conditions outlined above – the incapable and the transient – are often relevant to the perception of people with a terminal illness [11,12]. As a patient's health deteriorates, their health care needs necessarily increase and they may be too sick to repay a social debt. Outside health care settings, the healthy might see a foreshortened life as too great a risk for investment and socially exclude the terminally ill on that basis [5]. Obviously, PLWHA at the asymptomatic stages of their illness and those who are on antiretroviral therapies (ART) are better able to hide their illness status and delay social exclusion as they are still living healthy and productive lives with no visible signs of illness. However, regardless of whether or not a patient's illness is visible, the social exclusion judgements associated with conditions two and three above should be irrelevant to people working in health care settings by virtue of the fact that health professionals are trained to take care of sick people. In Thailand, there have been a number of ethical policies set out by the government and health care professional boards that prohibit health professionals from excluding PLWHA from health services on the basis of the above conditions. These include *The National AIDS Plan 1997/2001 *by the National AIDS Prevention and Control Committee, *The Declaration on the Rights of the Patients *1998 jointly issued by the Ministry of Health, the Council for Medical Doctors, the Council for Medical Nurses and the Council for Dentists, and *The Guideline on AIDS for Medical Doctors 2002 *by the Medical Council of Thailand.

The interest of this paper however is on the first condition – the 'cheater'. In thinking about people who are ill, the issue of cheating does not usually resonate because the illness status often casts the patient into the role of the *incapable *instead of the role of the deliberate non-reciprocator. Unlike many health conditions however, outside sub-Saharan Africa HIV/AIDS has typically been a disease of marginalised groups including men who have sex with men (MSM), injecting drug users and ethnic minority groups [13,14]. As a result, the disease stigma of HIV/AIDS is layered upon the co-stigmas associated with these socially marginalised high risk groups [15,16]. The issue of layered stigma is of particular importance in a country like Thailand where injecting drug use (IDU) is such a significant contributor of the local HIV/AIDS epidemic.

Despite the country's internationally recognised successes in preventing the spread of HIV/AIDS in the general population, there has been a rise in the prevalence and incidence of HIV amongst injecting drug users [17-19]. Whereas the annual incidence of HIV notifications in Thailand has declined from 143,000 in 1991 to approximately 19,000 in 2003 from 1991 to 1997, the percentage of seropositive men who had a history of IDU rose from 1% to 25%. By 2003, injecting drug users made up 33% of HIV prevalence in Thailand [18]. The marginalisation of injecting drug users is not only expressed in terms of the lack of targeted HIV prevention strategies for this high risk group, with recent studies also showing that PLWHA with a drug history are also often excluded from free ART [20]. However, it should be noted that the exclusion is also observed in many other countries including Russia, Lithuania and Malaysia [20,21].

A lack of government investment in harm reduction for HIV and other blood borne virus (BBV) prevention in Thailand and the paucity of health service targeted towards injecting drug users in general brings the notion of the drug user as a 'cheater' into question. One might argue that injecting drug users are in receipt of very little of society's resources and thus there is a limited social debt to which drug users are obligated to reciprocate. Although there is a clear societal cost associated with problematic drug use, the cost can be exacerbated when there is lack of government investment in ameliorating risk environments. For example, many studies have demonstrated the utility of a needle and syringe exchange programs in limiting BBV transmission among injecting drug users [e.g. [22]]. Drawing upon notions of governmentality theory and liberal individualism, Moore [23] identified injecting drug users (in the Australian context) as a population affected by a shift to neo-liberal governmentality. Rather than governments providing what historically might be considered a form a 'pastoral care' through the provision of expert care and services, obligations have shifted to the citizen as a rational and reflexive entity capable of making 'healthy' choices. This notion of the rational healthy consumer does not acknowledge the constraints on 'choice' that occur when government services are inadequate, or, in the case of drug use, the constraints on 'choice' restricted by the nature of addiction.

The lack of investment in HIV prevention and treatment for injecting drug users in Thailand has been linked to the country's long standing marginalisation of this subpopulation [24-26]. Recently, the social stigmatisation and exclusion of illicit drug users reached a new high following the popular 'War on Drugs' strategy of 2003–4 [27]. Announced in January 2003, under the strategy, persons connected to illicit drugs were portrayed as threats to the wellbeing and security of the nation and its people. As the most extreme form of social exclusion, an estimated 2,275 extrajudicial killing were carried out in the first three months of the campaign [28] and 73,231 arrests of suspected illicit drug traffickers and dealers were made by July 2003 [29].

A number of authors have predicted the negative impact the 'War on Drugs' policy might have on the stigmatisation and discrimination of illicit drug users in general and HIV prevention amongst injectors more specifically [18,28,30]. Unfortunately, there is currently a paucity of in-depth studies on the effects of IDU stigma on health personnel's perception of PLWHA. The centrality of the matter is shown in two recent quantitative papers which reported that avoidance attitudes by Thai nurses towards injecting drug users significantly exceed the avoidance attitudes towards PLWHA [31,32]. These findings raise questions about whether negative attitudes towards IDU could negatively impact upon the willingness of health personnel to care for PLWHA with an injecting drug history. The findings also pose a challenge to the conventional approach to HIV/AIDS stigma intervention which focuses almost exclusively upon the disease stigma of HIV/AIDS while neglecting the stigma of high risk behaviours such as IDU [see also [33]]. The more recent of the two studies [i.e., [31]] collected previously unreported qualitative data from interviews that explored the reasons behind the attitudes of Thai nurses towards injecting drug users and PLWHA. An exploration of the nurses' narratives could help explain the relative levels of avoidant attitudes towards PLWHA and IDU reported in previous publications, and provide new insights for addressing stigmatising attitudes by health personnel towards PLWHA.

Drawing upon this previously unreported narrative data, this paper aims to use the idea of social reciprocity to explore these nurses' views of injecting drug users and PLWHA. Specifically, the paper will explore whether or not and how the perceived willingness and ability of injecting drug users to engage in the social process of reciprocity might influence the marginalisation of PLWHA. The analysis is divided in four parts. First, participants' views on PLWHA divorced from views regarding the mode of transmission will be presented. This is followed by an analysis of their constructions of the illicit drug user in the absence of HIV. Overlaps between the two constructs and the issue of the HIV positive drug user are then assessed. Finally, an examination of the nurses' views on how the stigmatised might go about rectifying their social debt is presented. The paper will conclude with recommendations about approaches for stigma reduction.

## Methods

Twenty face-to-face semi-structured interviews were conducted with nurses recruited through a Bangkok nursing college. Half of the sample consisted of trainee nurses – final year undergraduates nursing students who had undergone various clinical rotations as part of their nursing training. The other half of the sample was made up of qualified nurses who, after years of clinical experiences, took up postgraduate training at the time of the research. The choice of a sample consisting of trainee as well as qualified nurses was partially determined by the quantitative component of the study in which qualification was treated as a co-variate for the analysis of avoidant attitudes [see [31]] for details of the quantitative component of the research method). The other reason for this sampling was so to ensure the sample covered a range of nurses at different stages of their careers and professional experiences.

The mean age of participants was 27 years (range = 21–44); 14 were female. Clinical experience amongst the participants ranged from four to 20 years. With the exception of two participants, all had experience caring for patients with HIV/AIDS. Results of the quantitative analysis showed that attitudinal responses did not significantly differ between trainee and qualified nurses [see [31]].

Participants were recruited via announcements made at the end of lectures by a Research Assistant not connected to the curriculum. Volunteers for the study were asked to write down their names and contact details on a piece of paper placed in the front of the lecture room. The first twenty names were selected, with researchers mindful of ensuring a gender ratio in the sample that reflected that of the year levels participants were recruited from.

Participants were first asked to complete a quantitative measure of stigma [see [31] for detail]. This was followed by in-depth interviews which explored participants' reasoning for their recorded attitudes, and more generally, their views towards HIV/AIDS and risk behaviours associated with HIV/AIDS. Examples of the interview questions are: What does HIV/AIDS mean to you and why? Upon learning that a person/patient is HIV positive, what is the first thing you think about and why? How do you feel about the HIV positive patients you have come across professionally and/or in your personal life? How do you feel about people/patients with HIV/AIDS and why? How do you feel about people who inject illicit drugs and why? Wherever possible, participants were asked to draw upon their experiences (personal/professional) in their reply to the questions.

The interviews were conducted in Thai by a local research assistant who had undergone two weeks of intensive training by the named researchers on this paper to conduct the interviews. The interviews were conducted between August and September 2004, during the second phase of the War on Drugs strategy. All interviews were audio-taped, transcribed in full and then translated into English. Pseudonyms were assigned to each interviewee and the name of the nursing college remains anonymous. The analysis of the data was conducted principally using the English transcripts by the first author. Transcripts were coded using NVivo software for analysing qualitative data and capable of providing basic quantitative analysis. The data were further explored using content analysis for the exploration of recurring themes. The analyses were shared and discussed between the three authors.

The study was reviewed and approved by Deakin University Human Research Ethics Committee, and is in strict compliance with the Helsinki Declaration.

## Results and discussion

### HIV in the absence of IDU

Participants made active efforts to portray PLWHA in a positive light. All participants denied at some point during the interview that they had viewed or treated PLWHA "differently" from other patients. Nonetheless, fear of the physical signs of AIDS (e.g. physical deterioration and the appearance of Kaposi's sarcoma) and fear of infection were commonly expressed. For example, as 'Wirat' commented, " [m]ost of them [PLWHA] are frightening because they are not lively, not fresh, ... gaunt, and pale".

The chronic physical suffering elicited sympathy and patients were "piti [ed] because... [of their] terrible condition [s]". The exclusion of PLWHA by the wider society also elicited a sense of injustice amongst some of the nurses. In describing a NGO meeting between PLWHA and seronegative people, that 'Samorn' helped to organise as part of her work, she said:

If they [seronegatives] see HIV positive people sit [ting] at the back [of the bus], the normal people [sic] will sit in the front. From my experience, ... no matter what they [PLWHA] did..., they would never get accepted.

Many participants also expressed frustration at the wider community's perception that the lives of PLWHA "have no value", and believed that the intolerance towards PLWHA was driven largely by fear arising from a lack of basic knowledge about HIV transmission. Moreover, a number of the participants stressed that the long asymptomatic period of HIV/AIDS meant that, given proper care, PLWHA could live "longer" and more fulfilling lives.

AIDS is ... destructive but if patients know to take care of themselves, it will be all right. People can use their knowledge and ability to continue their work. ('Rudee')

The nurses recognised the capacity of PLWHA to continue to contribute to society and, by implication, reciprocate the social benefits they received through community membership. The general community's intolerance towards PLWHA was therefore regarded as both obstructive to the well-being of PLWHA and interests of the community by preventing otherwise able and willing individuals from making positive social contributions. In order to assist PLWHA to live as constructive members of society, the nurses believed that they must be given proper instructions in terms of self care as well as understanding and encouragement to counter the prejudices they face. A large majority of nurses saw that it was within their professional duties to provide PLWHA with assistance.

As a nurse I have to look after them [AIDS patients]. As we know, in our society there are a lot of people who do not understand the patients, not even their relatives. It seems they have no value. I think I can support them by giving them advice, encouragement and care. ('Juaa')

I would be willing to help them [PLWHA] as much as possible, in health promotion or providing health education to their family... ('Wattana')

In the above construction, PWLHA as people divorced from a route of HIV transmission were spoken of by the nurses as people worthy of an investment of their professional time and effort. They were also explicitly portrayed as people able to contribute back to society. In addition, these participants perceived part of their role as contributing to the amelioration of intolerance of PLWHA by family and community.

### Views of illicit drug users in the absence of HIV

While the nurses were reluctant to express any negative opinions about PLWHA, such reservation was not observed in the attitudes expressed about injecting drug users, or drug users in general. When asked if they were more willing to accept PLWHA or drug users as a member of their social circles, few participants showed a preference for the injecting drug user. This finding is entirely consistent with those of the larger attitudinal survey that showed avoidant attitudes towards injecting drug users were significantly stronger than those towards PLWHA [31,32].

### Illicit drug users as 'cheaters'

Across the interviews, the moral condemnation of illicit drug use was expressed through descriptions of the behaviour as "bad", "unacceptable", "illegal" or simply "wrong". In this sense, drug users were perceived as ill-suited for reciprocal exchange because they were seen as likely to repay kindness by engaging in an activity that harmed the community not simply the individual. The perceived deliberate nature of this social harm potentially cast drug users as a social 'cheater'. However, the nurse participants never described the macro-, meso- and micro-factors affecting drug use or risky drug using behaviours such as sharing injecting equipment, or the complex interplay between these factors. Instead, drug use was caste as a freely made individual choice. These perceptions from nurses reconciles with the contention of Moore [23] and others [e.g. [34]] of a contemporary shift in public health discourse away from State responsibilities for facilitating healthy choices towards personal responsibilities and obligations for maintaining health. These perceptions necessarily made the drug user as directly responsible and blameworthy, with many participants cast unsafe injection practices as expressions of personal recklessness. For example, one participant explained, "...some drug users use the same syringe and get infected. They get addicted and don't fear infection..." ('Apinya'). And another, it is " [t]heir fearlessness led them to try out and engage in risky behaviours. ...It is like disrespecting their life and family... It's a self-centred approach." ('Samorn').

The image of the irresponsible drug user was further elaborated by 'Akara', a nurse with more than 20 years of experience,

[Drug users are t]hose who don't love themselves, lose self-control, and are drug-dependent. This is [true] for both male and female users. ... [drug use] is illegal...

In addition to a lack of self respect and will, the list of personal failings also extended to a failure of self-care, and drug users were commonly described as people who were "dirty" and "d [idn]'t learn to take care of their health". Studies have shown that health is a crucial factor in ones ability to engage in reciprocity (Point 2 and Point 3), and thus, staying healthy is identified as a means of protecting one's place in society [e.g. [35]]. While being ill reduces the ability to contribute to society in general, this notion was only apparent in participant narratives of PLWHA and not for drug users. The difference was at least in part due to the perception of differences in intentions between the two groups. As one participant explained,

AIDS patients may not get the disease intentionally. But drug use is not good. The users know that it is not good because it may affect their body, causing confusion and dizziness. ('Siriporn')

In this sense drug users were seen as bringing sickness upon themselves leading to self-exclusion from reciprocal social and moral obligations. In the absence of any acknowledgement of State responsibilities in creating environments that provide injecting drug users a choice to reduce risk, the presumed deliberateness of such behaviours makes the moral failures of the drug user more condemnable than PLWHA. The relative stigmatisation between PLWHA and injecting drug users is illustrated in the following dialogue.

Interviewer: ...as a nurse, you will not accept drugs. Why...?

'Samorn': Because, you know, drugs are bad.

Interviewer: No, I don't know. Assume that I am a foreigner who cannot understand Thai society.

'Samorn': Because, we [nurses] think that drugs are bad and stigmatise those who still take it. The level of honesty and openness of the view expressed was interesting within a culture that emphasises the saving of 'face' during social interaction, and sanctions those who violate the rule of face interaction by openly criticising others [36,37]. The openness might in fact indicate the severity of the IDU stigma, as such the open stigmatisation of illicit drug users was seen as morally correct and socially demanded as a form of social control of deviance.

Unlike the social marginalisation of people on the basis of serostatus, the nurses appeared to side with the view of the wider community. Whereas judgements within society about which groups become socially excluded (and therefore who is socially included) are sometimes considered arbitrary, the nurses in this study were often able to base their opinions of IDU and PLWHA on firmly held beliefs regarding the attribution of blame. The extent to which this might reflect the broader 'War on Drugs' policy and rhetoric pervading Thailand at the time of interviews is unknown. The difference in the nurses' judgements between the two groups might also be consistent with Buddhist precepts which emphasise showing kindness to the sick (e.g. as evident in the role Buddhist monks have played in caring for PLWHA) while deeming many of the high risk behaviours associated with HIV transmission as immoral and dishonourable [38].

Alarmingly, the above exchange portrayed the stigmatisation of drug users as a professional duty, one which nurses knowingly and actively endorsed. This understanding of nurses' professional duties is in direct conflict with the non-discrimination standard of health service delivery for all patients guaranteed by *The Declaration of Rights of the Patients *(1998), jointly issued by the Ministry of Health, the Council for Medical Nurses and the Council for Dentist . If this perceived collective responsibility is representative of the views of Thai nurses at the time of the study, it would indicate not only the intensity of the marginalisation faced by drug users, but suggests a troubling confusion over the nurses' understanding of their professional duties versus their duties as Thai citizens.

### Illicit drug use as a drain on community

Drug users were marginalised not only on the basis that they were 'cheaters' in reciprocal exchange. From the narratives of the nurses, the social exclusion of illicit drug users also stemmed from the quantum of the net drain they posed upon social goods. There are several ways in which this drain occurs. First, as established earlier, illicit drugs were seen as capable of destroying the minds and bodies of their consumers. Disabled bodies and minds make individuals unfit for social participation. As illustrated earlier, the fact that drug users were seen as having *chosen *to engage in behaviours that damage their health only made them less excusable. Self-destruction implies a loss of potential contribution to the collective resources, and a waste of resource potential. As 'Akara' observed,

... the money [used to buy drugs] should be used for other purposes. Instead they use drugs to kill themselves.

Second, treatment for drug addiction, while potentially preventing further resource allocation to drug users, also drains valuable social resources from the community. Whereas the treatment of PLWHA at the final stage of their illness (when they can no longer reciprocate) requires expenditure, it is a confined investment as "death is predictable" and effectively puts a cap on treatment expenditure. In contrast, the investment in treatments for drug dependence could be long term and continuous. Third, as the quotes below illustrate, nurses believed that illicit drugs make drug users 'unpredictable' and 'dangerous' to others. This makes drug users liabilities to public safety. Participants linked drug use to violence and crimes ranging from petty theft to murder. Many expressed genuine fears towards drug users.

if he [a drug user] used a lot of drugs, he might have delusions and hurt me. ('Juaa')

...Some drug users cause general people to die. After they become conscious, they cannot remember what they had done. ('Teerasak')

The cost to the community thus extends beyond the inability of the individual to contribute to community resources. As such, the 'dangerous' behaviours of the 'unpredictable' drug user have the potential to further burden the community by interfering with the physical safety of others. As illustrated by the following, society and families are also perceived to be burdened with financial vulnerabilities associated drug use:

... drugs destroy... not only health but ... [e]ntire families. There will be financial problems... ('Akara')

...drugs are related to many problems including theft, even stealing from one's own parents. Everything is lost. ('Siriporn')

Furthermore, as 'mad', 'violent' drug criminals were unable to reach their full potentials, they were also deemed unfit to assist in the development of the nation. On a societal level, illicit drugs were thus cast as a social, economic and 'national security' threat. As 'Malee', a nurse of more than ten years, explained:

... [drugs] destroy national security...it destroys the brain and thoughts. It changes people and makes them do bad things. It affects youth especially as they may not be able to study. Other people [drug users] may steal, and not use their brain to further develop the country.

Some participants viewed drug use as a more serious socio-economic problem than HIV/AIDS, thus partially explaining why the former was significantly more stigmatised than the latter. As 'Rudee' explained,

... [drug creates] ...economic problems ... it destroys the population in the country and reduces the quality of the population .... AIDS is also destructive but if patients know to take care of themselves, it will be all right. People [with AIDS] can use their knowledge and ability to continue their work.

Further contributing to social exclusion, the stigma of drug user was also seen as 'contagious'. Given the illegality and public hostility towards drug use, some participants believed that they would be 'tainted' simply by becoming associated with drug users. In this regard, the stigma of drug use was seen as contagious. As 'Akara' explained,

If I associate with drug users, I will also be at risk [of being] put in jail. ...other people will see me in a negative manner and assume that I also take, sell and distribute drugs...

It is worth noting that the generalised image of the drug user or addict as a threat to personal and public safety draws upon the side effects of a wide variety of illicit drugs, many of which were not historically connected to HIV transmission because the substances could not be or were not commonly taken intravenously. The most common illicit drug taken intravenously in Thailand was heroin [39,40], a depressant not commonly known for causing hallucination or delusions. Drugs more known for producing these side effects in a minority of users belong to the stimulant and hallucinogens categories (e.g. amphetamines, MDMA). Coincidently, it was concerns over the increase in the prevalence of amphetamine type substances (e.g. 'ya baa' – literally translated as 'crazy medicine' [41] that was largely responsible for inspiring the 'War on Drugs' policy [29].

The generalised image of drug users presented by nurses was somewhat surprising given that it was made clear to participants that the focus was their attitudes toward injecting drug users because of the direct link between unsafe injection and HIV transmission. It was unclear from the interviews whether generalisations were indicative of a lack of knowledge about the variety of illicit drugs available in the market and their different psychoactive effects. From the quotes provided earlier, it appears that this demonised stereotypic image of the drug addict was held also by nurses who acknowledged that antisocial behaviours in the most extreme forms occurred only to a minority of users. Furthermore, the ways in which drug use was linked to macro socio-economic and national security issues echoed the message of the 'War on Drugs' campaign, which evoked such notions repeatedly. To our knowledge, the effects of the 'War on Drugs' campaign on health care workers' attitudes about drug using patients and their effects on care provision have not been investigated.

### The layering of the social exclusions of PLWHA and drug users

While many participants made active efforts to separate their opinions of PLWHA from drug users, even from some of the quotes presented earlier, the distinction was often blurred. Indeed, all participants viewed IDU as a common direct cause of HIV infection, and since individuals are assumed to have engaged in this co-stigmatised behaviours by choice, PLWHA were sometimes logically blamed for their illnesses.

AIDS tends to be related to behaviour. ... If people don't have risky behaviour, illness will not visit them. ('Wichar')

AIDS relates to behaviour. Most people are infected via improper behaviours. ('Sangwaan')

Furthermore, while most participants expressed a willingness to help PLWHA on both a personal and professional level, many felt that " [i]t is different that they are related to bad behaviour" ('Sangwaan').

This perceived causal relationship between the choice to engage in high risk behaviours and HIV infection as the logical outcome also resulted in some layering in the dislike of PLWHA and the dislike of illicit drug users. As one participant ('Dusit') stated,

...The reason why I don't like AIDS is... the reason why I am least willing to associate with a drug user.

Thus, in so far as HIV/AIDS is constructed as a natural outcome of immoral behaviours, the presence of the illness becomes an obvious marker of the presence of those behaviours. PLWHA were therefore not judged so much for their chronic illness, but for their assumed or actual risk behaviours. In this regard, the stigmatisation of HIV/AIDS is inseparable from the stigmatisation of IDU. It would be however incorrect to claim that participants had labelled all PLWHA as drug users and/or engaged in other marginalised behaviours associated with HIV transmission. Many participants had emphasised that, through their clinical training and experience, they had arrived at the understanding that not all PLWHA were infected through disreputable means. As 'Siriporn', a fourth year nursing trainee who was working in a paediatric unit explained,

AIDS patients like child patients who have got AIDS from their parents, or housewives that have got AIDS from their husband have got it unintentionally.

Indeed, many interviewees considered their understanding that not all PLWHA were guilty of marginalised risk behaviours as a key difference between the professional nurse and the lay public. As 'Krasin', another fourth year nursing trainee working at a psychiatric unit explained,

In society, people in general don't know about the disease (AIDS). They think they will not get it because they aren't promiscuous.

The dichotomised constructions of patients into guilty versus innocent victims of AIDS has already been widely reported in the HIV/AIDS literature [e.g. [16,42]]. In this study, many of the nurses also admitted that their sympathy towards PLWHA was largely a function of patients' perceived guilt. For example,

Those infected by their boyfriends or girlfriends are more pitiful than those infected from sex workers and drug use. ('Wattana')

Since the mode of transmission of the patient is not always known to nurses, the distinction between 'guilty' and 'innocent' PLWHA is often arbitrary at best. This could result in some PLWHA being wrongly associated with co-stigmatised behaviours. However, this same interviewee was also quick to add that personal prejudices did not affect the quality of care they provide to patient with HIV/AIDS,

However, we give them the same treatment. We have to know the cause of infection. But I feel negatively to those who used to take drugs and visit sex workers. ('Wattana')

In spite of this disclaimer which was also used by a number of the other interviewees, it is unclear whether all participants were in fact able to separate their personal biases in practice. The potential confounding overlap between the attitudes of the professional and social self among nurses is somewhat illustrated by the apparent contradictory nature of the above response. While proclaiming that patients with HIV/AIDS were all treated the same, the cause of infection was something that nurses were entitled to know. How notions of universal standards of care could be reconciled with needing to know the mode of transmission and the admission of "feel [ing] negatively" towards drug users and visitors of sex workers was not resolved. The separation of the guilty and innocent victim and the notion of universal care seems dubious especially given that there was a general sentiment amongst the participants that, " [i]f people get infected and behave badly, they should not be helped" ('Malee').

### Redress for the HIV positive 'cheater'

To become worthy of assistance, some nurses believed that the HIV positive drug user had a duty to redeem their moral failures through character reform. As such, the outcast must reinitiate the reciprocal exchange between the individual and the community. To do that, s/he must first give up the behaviour(s) that caused their infection. Continuous drug consumption posed an ongoing drain on social resources not only because intoxication made drug users physically unfit to fulfil their citizen duties, but continued engagement in unsafe injection also made the seropositive drug user a vector for spreading the virus to others. Abstinence from drug use thus offered a symbolic gesture, illustrating that the former drug user was capable of fulfilling some of the demands of their citizenships. Without this gesture, societal investment would be wasteful.

...if they [HIV positive drug users] can change their behaviour, I cannot see why society must reject them. If they behave the same, they are blamed. ('Teerasak')

To some of the participants, abstaining from illicit drugs was insufficient. Abstinence only prevents a future impost on social resources by prospectively preventing the direct harms of drugs and preventing further infectious disease transmission. Abstinence however does not address the already accumulated costs to the community. To redeem past sins, the former addict also has a social obligation to serve as a role model for others engaged in co-stigmatising behaviours but not yet infected with HIV. As such, former drug users already infected with HIV/AIDS have a duty to actively engage in health promotion.

... if ... [PLWHA] behave well, and help society; society should provide them with opportunities. [Behaving well]... means not transmitting one's disease to others; being a model for others to prevent infection; and making people know about the dangers of AIDS... how it affects health, society and [the] nation. Behaving well [means], not taking drugs and not visiting sex workers – [this] can be done even among AIDS patients. ('Malee')

While some of the other nurses might not have articulated the issue of reciprocal exchange as explicitly as 'Malee', similar themes were covered:

...If people know what diseases they have but still don't change their behaviour in such a way to protect and take care of themselves, they will be the same. This is because their behaviour is a part of helping them to have better health and fewer risk factors. ('Rudee')

...At the first week during my study here, there was a person... [who] was HIV infected but could lead his live and did useful activities in the society. I would like him to be a model for other infected people or AIDS patients who are still strong and can do things. ('Sangwaan')

The logic presented here fails to acknowledge the socio-structural and biological factors associated with the uptake of drug and addiction. This reciprocal approach is somewhat of a catch-22 for someone addicted to drugs. Before being able to contribute to society a dependent injecting drug users would likely require medical treatment and perhaps some redress of the socio-environmental factors that predispose them to illicit drug consumption [18,24]. The manifestation of "behav [ing] well, and help [ing] society" in terms of not "transmitting one's disease" and "not taking drugs" is predicated (or most likely) under conditions where adequate services are provided by the community (e.g. access to clean needles and syringes and dependence treatment). Yet, disqualified from social support, the nurses' attitudes suggest the drug users are expected to rely on will alone to actualise their redemption and rejoin society before they are worthy of such support or investment. This again reflects a neo-governmentality discourse and evokes notions of the self-actualising, reflective and rational citizen. This reasoning is also contradictory to the nurses' own understanding presented earlier that describes a lack of will (perhaps the only implicit acknowledgement by nurses of the nature of addiction) as a key factor leading to illicit drug consumption in the first place. The logic of redemption also illustrates the intricate link between the stigmatisation of PLWHA and the stigmatisation of high risk behaviours. While drug users are openly stigmatised, PLWHA are still described as valuable citizens. However, unlike the seronegative public whose social values are automatically credited, the social value of the seropositives must be continuously and actively validated by PLWHA, or they risk bearing the stigma of associated high risk behaviours.

## Conclusion

Using the concept of reciprocity, this paper illustrates the layering between the social stigmatisation and exclusion of PLWHA and illicit drug users within the narratives of a group of Thai nurses. The various reasons underlying the lack of support for illicit drug users are explained in terms of the drug users' perceived willingness and ability to engage in social reciprocity. While it should be noted that there were variations in participants' biases towards illicit drug users, illicit drug use was largely portrayed as a deliberate moral failure of the individual, making illicit drug users 'cheaters' in the larger social system valued by participants. The findings of the study show that the perceived burden drug users represent to the social system was extensive. As such, illicit drug use was constructed not only as a threat to individual health, but the safety and wellbeing of the nation and its citizens. The social stigmatisation and exclusion of drug users were therefore seen as a necessity for the greater good and survival of the community as a whole. While the nurses might have expressed their biases towards drug users out of a sense of collective duty, such attitudes clearly conflict with their professional obligation to provide equitable care to all patients, including those with a history of drug use.

Conventional approaches to HIV/AIDS stigma intervention have typically focused on reducing negative attitudes towards the disease while neglecting the negative views associated with high risk behaviours [43,44]. The narratives presented in this paper illustrates that the social stigmatisation and exclusion of illicit drug users also has important implications for nurses' views of PLWHA, and therefore should not be overlooked in future stigma interventions. Despite kind words being expressed by participants that illustrated a level of relative acceptance towards PLWHA, the acceptance was conditional on patients' ability to prove their 'innocence' from high risk stigmatised behaviours. Not only does this condition pose obvious problems for PLWHA who had engaged in high risk behaviours, those innocent of such behaviours are also burdened with the difficulty of proving their 'innocence' in practice. Such discriminatory attitudes raise issues of professional ethics. Although some nurses had denied acting out of their prejudices, the gravity and sometimes contradictory nature of the prejudicial responses expressed suggest that this disclaimer may not always apply in practice. Further studies should look at ways of understanding the relationships between nurses' prejudicial attitudes and their actual professional behaviours to PLWHA and to people with a history of associated co-stigmatised behaviours.

Since the stigmatisation of PLWHA can largely be traced to the stigmatisation of high risk behaviours including drug use, it seems logical that reducing the stigma of those behaviours would be crucial to lowering the stigma of PLWHA. Decreasing stigma requires improvements in the social status of the stigmatised. From the data in this study, it seems that the low social status of drug user was derived from what might be considered an unfair and non-representative image of this group. Furthermore, the position by which individuals were blamed for their drug addiction and HIV infection was assisted by an apparent ignorance of the complexity of the socio-political and epidemiological factors related to illicit drug use, addiction and the agency of the individual to up-take harm minimisation strategies. To remove this blame, alternative ways of constructing and understanding illicit drug use, risk environments and risk behaviours should be incorporated into the nursing curriculum. For example, increasing nurses' understanding of distal socio-economic and political factors concerning illicit drugs and related BBV epidemics could also help contextualise the uptake of illicit drugs and ease the blame on individual users. Likewise, providing nurses with knowledge of harm reduction and structural issues that affect an individual's ability to reduce their risk of harm could also alleviate the attributed guilt of the seropositive drug user. Modifying the focus of nursing curriculum in the suggested ways might be difficult given the current hostile political climate surrounding illicit drug use in Thailand. Redirecting government policy towards embracing a more general harm reduction framework on illicit drugs might thus be necessary to provide an environment for such changes to occur.

## Abbreviations

Acquired Immune Deficiency Syndrome: AIDS; Human immunodeficiency virus: HIV; Injecting drug use: IDU;  Patients living with HIV/AIDS: PLWHA.

## Competing interests

The authors declare that they have no competing interests.

## Authors' contributions

KYC conceived of the study and was principally responsible for the study design, the overseeing the data collection, data analysis and the drafting of the manuscript. MS contributed to the data analysis and the drafting of the manuscript. DDR contributed to the initial conception of the study, data analysis and the drafting of the manuscript. All authors have read and approved the final manuscript.
